# Inpatient care provider perspectives on the development and implementation of an addiction medicine consultation service in a small urban setting

**DOI:** 10.1186/s13011-022-00497-9

**Published:** 2022-10-27

**Authors:** Madelaine Beckett, Ramm Hering, Karen Urbanoski

**Affiliations:** 1 Department of Medicine, University of British Columbia, Vancouver, BC Canada; 2 Island Health, Victoria, BC Canada; 3 Canadian Institute for Substance Use Research, University of Victoria, 2300 McKenzie Ave, Victoria, BC V8P 5C2 Canada; 4 Public Health and Social Policy, University of Victoria, Victoria, BC Canada

**Keywords:** Addiction medicine consult, Inpatient care, Care quality, Survey research

## Abstract

**Background:**

To evaluate provider perspectives on the development and implementation of an inpatient Addiction Medicine Consult Service, including their awareness of the service, its perceived role in the continuum of care, and changes over time in their perceptions of care quality for inpatients with substance use disorders.

**Methods:**

Repeated cross-sectional survey of hospital-based physicians, nurses and social workers performed at service launch (April–June, 2017) and 4 years later (March–June, 2021).

**Results:**

Providers had generally positive perceptions of the service and its impact on care quality, but encountered significant barriers at both time points in meeting patient needs (related to high patient complexity and difficulty connecting patients with community services post-discharge). Relative to physicians and social workers, nurses were less likely to be familiar with the service or see it as beneficial.

**Conclusions:**

Findings indicate that the service fills a gap that existed previously in the local system of care; however, numerous opportunities exist to further strengthen the system beyond the hospital setting to promote longer-term health among people who use substances. For nurses in particular, outreach, education, and other resources (e.g., dedicated nursing role support, nurse liaison) are warranted to ensure that nurses feel supported and confident caring for this patient population.

## Background

Substance used disorder (SUD), defined as the persistent use of drugs, tobacco, or alcohol despite harmful consequences, commonly co-occurs alongside a range of health conditions [1, 2]. When people with SUD are hospitalized for other conditions, unaddressed SUD can negatively impact care and result in missed opportunities for engagement in substance use services [3–6]. Epidemiological data show that SUD are a major healthcare challenge, with dramatic rise in mortality since 2016 and complications of SUD now the fourth most common cause of hospitalization in Canada in 2020–2021 [7, 8]. Yet medical education in Canada and elsewhere offers little training to physicians in substance use and related harms, such that many report a lack of comfort in the management of SUD [9–11]. Additionally, care for hospitalized patients with SUD has traditionally been directed only at the acute medical concern, lacking a holistic approach to address co-occurring social determinants of health that contribute to poor outcomes and representation to hospital [12]. Addiction Medicine Consult Services (AMCS) have emerged as an interdisciplinary response to supporting improved care quality for people with SUD in inpatient settings [13, 14].

AMCS teams are comprised of physicians with training in addiction medicine, often with inter-professional support from nurses and social workers, who offer on-call services for diagnosis, delivery of medical and psychosocial services, and discharge planning for patients in hospital settings [6, 15–18]. Teams may also offer education to inpatient care providers on addiction medicine and shape related hospital guidelines and policy [13]. Evidence supports the feasibility and acceptability of inpatient AMCS [12, 19, 20], with improvements in care quality for inpatients with SUD [6], reduced readmissions and length of stay [13, 16], and improved discharge outcomes following implementation [6, 21–23].

In Canada, AMCS teams began to emerge in major urban centres a decade ago and are now being developed in smaller urban and suburban cities. Studies of the use and characteristics of AMCS support their continued integration into existing systems of care [6, 15–18]. This body of research largely relies on hospital records and administrative data documenting referrals, consultations, patient characteristics, and follow-up visits; with studies of patient-reported perceptions of care being less common [12, 20]. There is a particular gap in studies of provider perspectives of AMCS implementation and operation, including their impressions of the service, its role in the continuum of care, and impacts on care quality. This information is crucial to service design and to informing any adaptations required to overcome barriers and meet local need. The present study addresses this gap in the literature through an evaluation of provider perspectives on an AMCS, as it was being developed and implemented in a small urban area in Canada.

In 2017, a group of addiction medicine physicians established an AMCS in two general hospitals in Victoria, British Columbia, a small provincial capital city (est. population 2021 = 390,000). The team is staffed by nine rotating physicians certified in addiction medicine (primarily certified in family medicine or internal medicine), two social workers, and three peer support workers (people with lived experience of SUD) who work across the two hospitals. Physicians are scheduled a week at a time; each is therefore on service for 1 out of every 4–5 weeks. Continuity of care is promoted through extensive handover notes, with support from full-time social workers and peer support workers. All patients (in any setting in the two general hospitals) with known or suspected SUD are eligible for referral by their Most Responsible Physician. The AMCS provides a range of services to support patients with symptom navigation, treatment initiation, and discharge planning. Consults are conducted directly by the AMCS physicians and are documented in the electronic medical record. All requests for consults for SUD are accepted. Consults for patients with chronic pain are only accepted if they have concurrent SUD. The service operates Monday to Friday during daytime hours with in-person and telephone consultations. In its first 4 years of operation (June 2017 to March 2021), 4411 encounters with the AMCS were documented, for 2886 individual patients (unpublished data, Vancouver Island Health Authority).

A study was designed to coincide with the development and implementation of the AMCS in Victoria, British Columbia, to inform initial planning and evolution of the service over time. The objective was to evaluate provider awareness of the service, its perceived role in the continuum of care, and changes over time in their perceptions of care quality for inpatients with SUD.

## Methods

### Study design

We conducted repeated cross-sectional surveys of health care providers who work at the two hospitals where the AMCS operates. Providers were surveyed at two time points: in the 3 months leading up to the opening of the AMCS (April–June, 2017) and 4 years later (March–June, 2021). The second round of data collection took place in the context of public health restrictions and protocols designed to minimize the transmission of COVID-19. No changes to the study protocol were required, such that study procedures were the same across time points. A pragmatic repeat cross-sectional design was chosen as longitudinal study was felt to be limited by potential staff turnover and anticipated high attrition rate which would limit meaningful analysis.

Eligible participants were all nurses, physicians, and social workers who work in an inpatient setting at one of the two hospitals. The questionnaire was developed by the authors, based on prior experience with program evaluation and survey development, combined with clinical experience in AMCS elsewhere. Items were tailored to the three professions. Representatives from all provider groups provided feedback on early drafts of the questionnaire, which was incorporated into the final version. Topics assessed at both time points included the frequency of encountering people with SUD and related conditions in hospital (rated as unsure, never, sometimes, often), their experience of barriers encountered in care delivery (rated as not at all, somewhat, significant), and their perception of care quality (1 = lowest; 10 = highest). Providers were asked to rate their level of comfort delivering a range of interventions for people with SUD, with interventions matched to disciplinary care roles: physicians were asked about screening and pharmacological interventions (17 items); nurses were asked about management of withdrawal and co-occurring health conditions (6 items); and social workers were asked about connecting patients with community services (5 items). Items were rated on a 5-point scale (1 = very uncomfortable; 5 = very comfortable), and a weighted mean (i.e., the mean of answered items) was calculated to represent a summary measure of comfort with care management for inpatients with SUD. Internal consistency was good for the summary measures of comfort (Cronbach’s alpha = 0.89 for physicians and social workers, and 0.85 for nurses). The survey in 2021 also included items to assess awareness of and frequency of interaction with the AMCS, and its perceived impact on inpatient care for people with SUD.

At both time points, posters advertising the survey were placed in nursing stations, staff lounges, physician lounges, and other clinical areas. Information about completing the survey was also emailed out. Surveys were completed online through RedCap, an electronic data capture platform. All respondents provided implied consent prior to starting the survey. Respondents had the option of entering their email address into a draw for one of six $100 (CAD) gift certificates to a bookstore. Activities were approved by the Research Ethics Board at Island Health, the regional health authority (#J2016–119).

The survey was completed by 347 providers (183 in 2017 and 164 in 2021). 21 respondents were excluded because they did not meet the eligibility of the study (they self-reported working only in community settings). The final sample includes 326 respondents (168 in 2017 and 158 in 2021), with 92 physicians (62 in 2017; 30 in 2021), 184 nurses (80 in 2017; 104 in 2021), and 50 social workers (26 in 2017; 24 in 2021). A small number of respondents (*n* = 4) reported that they participated at both time points; however, the survey was designed to be cross-sectional at two time points and linkage over time of individual responses was not performed.

### Analysis

Descriptive statistics (proportions for categorical variables; mean and standard deviation for continuous variables) were calculated for provider perceptions of the AMCS (in 2021) and provider perceptions of care for inpatients with SUD (in 2017 and 2021). For provider perceptions of care, differences between 2017 and 2021 were assessed using chi-square tests for categorical variables (Fisher’s exact *p*-values are reported) and t-tests for continuous variables. All analyses were stratified by provider type. Quantitative analyses were conducted in Stata 16 using an alpha level of 0.05 to determine statistical significance. Responses to open-ended items (on potential improvements to the AMCS and additional barriers to managing SUD in hospital) were coded inductively to identify themes. Responses were copied into an Excel spreadsheet and grouped to identify conceptually similar ideas. Direct quotes are provided to illustrate key themes. Quotes are identified by provider type (P = physician, N = nurse, and SW = social worker) and survey year (2017, 2021) for context.

## Results

### Sample description

The study was successful in capturing respondents from diverse hospital settings, with some differences in sample composition across the two time points. Physician respondents were most likely to work in family medicine or as a hospitalist, relative to speciality areas such as psychiatry, internal medicine, surgery, or emergency medicine (Table 1). Medical wards (e.g., obstetric, cardiac, rehabilitation units) were also the most reported settings for respondents from nursing and social work. In 2021 (compared to 2017), fewer physicians had specialist training, fewer nurses reported working in psychiatric and emergency settings, and fewer social workers reported working in surgical or medical wards. All social workers had received some form of training in substance use; however, only a minority of nurses (30.0% [*n* = 24] in 2017 and 22.1% [*n* = 23] in 2021; Fishers exact *p* = .237) and physicians (33.9% [*n* = 21] in 2017 and 43.4% [*n* = 13] in 2021; Fischer’s exact *p* = .490) reported prior training in addiction medicine/substance use. Types of training ranged from electives and workshops to addiction medicine certification.

**Table 1:** Sample description (*n* = 326)

	2017 (***n*** = 168)		2021 (***n*** = 158)	
	n	%	n	%
**Physicians (*n*** **= 92)**	62		30	
Specialty				
Family medicine/ hospitalist	28	45.2	21	70.0
Psychiatry	12	19.4	2	6.7
Internal medicine	8	12.9	3	10.0
Emergency medicine	6	9.7	0	–
Surgery/anesthesiology	4	6.5	1	3.3
Other	4	6.5	3	10.0
**Nurses (*n*** **= 184)**	80		104	
Ward^a^				
Medicine	38	47.5	55	55.9
Surgical	30	37.5	42	40.4
Psychiatric	21	26.3	13	12.5
Emergency	17	21.3	13	12.5
Other	0	–	1	1.0
**Social workers (*n*** **= 50)**	26		24	
Ward^a^				
Medicine	19	73.1	16	66.7
Surgical	9	34.6	1	4.2
Psychiatric	9	34.6	8	33.3
Emergency	10	38.5	13	54.2
Other	5	19.2	3	12.5

^a^Respondents could name more than 1 setting (categories sum to more than 100%)

### Provider perceptions of the AMCS

The majority of respondents surveyed in 2021 were aware of the AMCS, including almost all of the physicians and social workers and three-quarters of nurses (Table 2). Most physicians had referred patients to the AMCS since it began in 2017, and two-thirds of social workers reported that they somewhat or very frequently interacted with the AMCS. In comparison, less than a third of nurses reported that they somewhat or very frequently interacted with the AMCS team. Of the three provider types, nurses were the least likely to report familiarity with the AMCS, with only 30.1% being somewhat or very familiar with the service (compared to 76.6% of physicians and 75.0% of social workers). Sizable minorities of physicians and nurses reported that they were very unfamiliar with it (20.0 and 33.0%, respectively). Most respondents perceived that the AMCS had a positive impact on inpatient care for SUD, however, nurses were least likely to indicate that the AMCS had a significant impact on care for SUD (52.4% vs. 96.7% of physicians and 79.2% of social workers) or that communication with the AMCS team was very helpful for patient care (50.0% vs. 86.7% of physicians and 79.2% of social workers).

**Table 2:** Provider perceptions of the AMCS (n = 158)

	Physicians		Nurses		Social Workers	
	2021 (***n*** = 30)		2021 (***n*** = 104)		2021 (n = 24)	
	n	%	n	%	n	%
Aware of the AMCS						
Yes	28	93.3	77	75.5	23	95.8
No/Unsure	2	6.7	25	24.5	1	4.2
Referred a patient to the AMCS:						
Yes	27	90.0				
No	3	10.0				
Frequency of interacting with the AMCS:						
Very infrequently			26	25.2	3	12.5
Somewhat infrequently			28	27.2	3	12.5
Neutral			18	17.5	2	8.3
Somewhat frequently			28	27.2	5	20.8
Very infrequently			3	2.9	11	45.8
Level of familiarity with the AMCS						
Very unfamiliar	6	20.0	34	33.0	2	8.3
Somewhat unfamiliar	1	3.3	28	27.2	3	12.5
Neutral	0	–	10	9.7	1	4.2
Somewhat familiar	4	13.3	25	24.3	8	33.3
Very familiar	19	63.3	6	5.8	10	41.7
Impact on support for treatment and care:						
Minimal	0	–	8	7.8	0	–
Neutral	1	3.3	41	38.8	5	20.8
Significant	29	96.7	54	52.4	19	79.2
Perceived helpfulness of communication with the AMCS team:						
Not helpful	0	–	5	4.9	0	–
Neutral	4	13.3	46	45.1	5	20.8
Very helpful	26	86.7	51	50.0	19	79.2

Respondents had the opportunity to provide suggestions for the further development and potential improvement of the AMCS. Responses to this open-ended item commonly involved requests for expanded hours of coverage (to include weekends, holidays, and after hours), and/or additional staffing. These suggestions were linked to ensuring that opportunities are not lost to engage with inpatients with SUD who may have difficulty staying in hospital (e.g., *“More staffing to ensure you are reaching as [many] patients as possible…” N-2021*). Several nurse respondents noted a need for greater visibility and information-sharing about the service (e.g., “*It would be beneficial to have an in-service [training] on how this service can be accessed and how they provide care. What types of discussions nurses should be having with their patients receiving this treatment.” N-2021*). Additional requests reflected an interest in more educational opportunities on SUD, how to meet patient needs, and treatment options. A final theme spoke to streamlining the process for requesting consults, including linkage with emergency departments so that consults can be arranged quickly and allowing requests for consultations from nurses in addition to physicians (e.g., *“Some way for flagging patients who present to emergency frequently/multiple admissions for addictions related issues in the past. That way AMCS can become involved quickly to help manage their care.” N-2021*).

### Provider perceptions of inpatient care for SUD

**Fig. 1:**
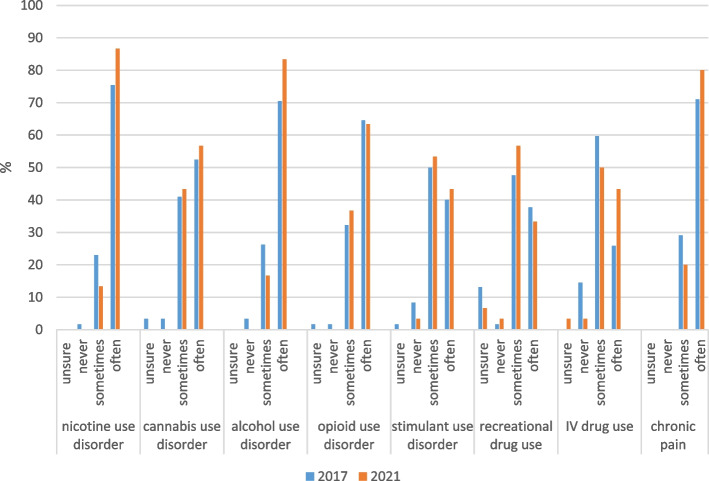
Frequency of encountering SUD and related conditions in inpatients, change over time among physicians (*n* = 92)^a^. ^a^Fisher’s exact tests were used to examine differences by year; all ns (*p* > .05)

**Fig. 2:**
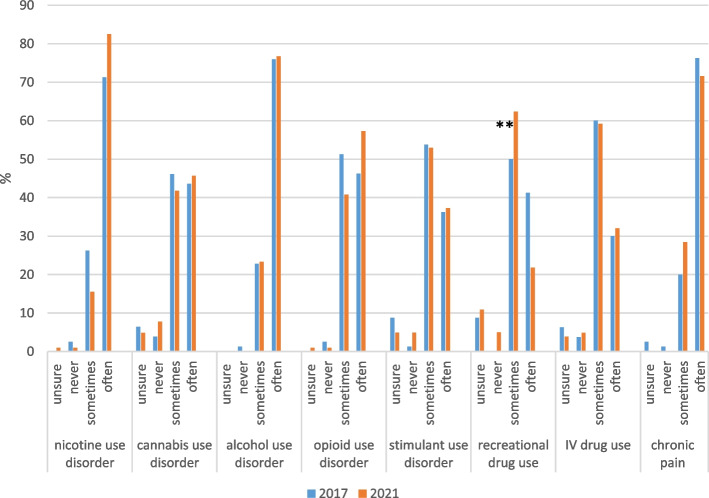
Frequency of encountering SUD in inpatients, change over time among nurses (n = 184)^a^. ^a^Fisher’s exact tests were used to examine differences by year. ** *p* < .01

At both time points, physician and nurse respondents reported that they commonly encountered SUD and related conditions among inpatients, particularly nicotine and alcohol use disorder and chronic pain (Figs. 1 and 2). A slight shift over time was apparent for frequency of encountering inpatients with intravenous (IV) drug use, with physicians being more likely to report often encountering IV drug use in their practice in 2021 (43.3% vs. 25.8% in 2017), although the difference was not statistically significant (Fisher’s exact *p* = .073). Nurses were significantly less likely to report encountering recreational drug use often in 2021 (21.8% vs. 41.3% in 2017; Fisher’s exact *p* = .010). The majority of social workers indicated that they often encounter SUD among inpatients, increasing from 69.2% in 2017 to 91.7% in 2021 (a non-significant change, Fisher’s exact *p* = .077). No respondents at either time point indicated that they never encountered SUD in their inpatients, or that they were unsure.

On average, physicians rated their level of comfort with SUD interventions as falling between neutral and somewhat comfortable (3–4 on a 5-point scale), with a slight but significant increase over time (Table 3). Physician perceptions of care quality increased considerably from 3.93 (*SD* = 2.02) to 7.09 (*SD* = 1.85) (on a 10-point scale). Mean ratings of comfort with SUD interventions among nurses and social workers were comparable to those of physicians, but with no change between 2017 and 2021. Nurse and social worker perceptions of care quality both increased significantly over time, although the magnitude of the change is relatively small (among nurses, from 4.68 (*SD =* 2.48) to 5.81 (*SD =* 2.27); among social workers, from 4.27 (*SD =* 2.31) to 5.72 (*SD =* 1.95)).

**Table 3:** Level of comfort with SUD interventions and perceptions of care quality (n = 326)

	2017				2021					
	n	Mean	SD	95% CI	n	Mean	SD	95% CI	t	p
Physicians										
Level of comfort with SUD interventions ^a^	62	3.25	0.64	3.09–3.41	30	3.70	0.59	3.48–3.92	−3.25	.002
Care quality ^b^	56	3.93	2.02	3.39–4.47	29	7.09	1.85	6.39–7.80	−7.03	<.001
Nurses										
Level of comfort in addressing SUD and related issues ^c^	80	3.22	0.72	3.06–3.38	103	3.42	0.79	3.26–3.57	−1.71	.089
Care quality ^b^	78	4.68	2.48	4.12–5.24	95	5.81	2.27	5.34–6.27	−3.10	.002
Social workers										
Level of comfort with SUD interventions ^d^	26	3.67	0.95	3.29–4.05	24	3.67	1.02	3.24–4.11	−0.02	.984
Care quality ^b^	22	4.27	2.31	3.25–5.30	22	5.72	1.95	4.85–6.58	−2.24	.030

^a^weighted mean of ratings of comfort delivering 17 interventions for SUD (1 = very uncomfortable; 5 = very comfortable)

^b^mean rating of perceived quality of care provided to people with SUD in inpatient settings (1 = lowest; 10 = highest)

^c^weighted mean of ratings of comfort delivering 6 interventions for SUD (1 = very comfortable; 5 = very comfortable)

^d^weighted mean of ratings of comfort delivering 5 interventions for SUD (1 = very comfortable; 5 = very comfortable)

**Fig. 3:**
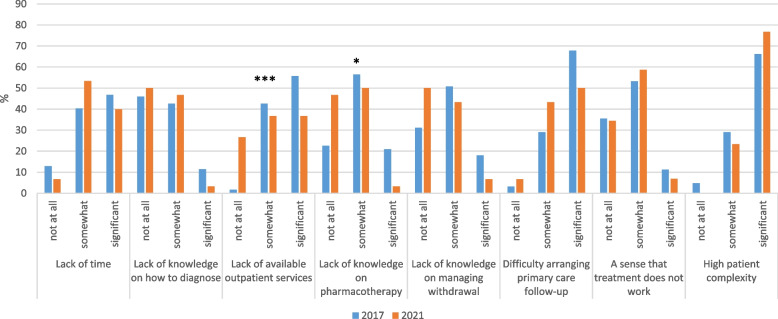
Barriers to caring for SUD in inpatients, change over time among physicians (*n* = 92)^a^. ^a^ Fisher’s exact tests were used to examine differences by year. * *p* < .05 . *** *p* < .001

Among physicians, high patient complexity was the most reported barrier to caring for SUD in inpatients, with 66.1 and 76.7% of physicians describing it as a significant barrier in 2017 and 2021, respectively (Fig. 3). This was followed by difficulty arranging primary care, reported to be a significant barrier by 67.7 and 50.0% of physicians. Neither of these barriers showed a change over time. In contrast, the proportion of physicians reporting a lack of available outpatient services as a significant barrier declined from 55.7% in 2017 to 36.7% in 2021 (Fischer’s exact *p* = .001), while the proportion reporting a lack of knowledge on pharmacotherapy as a significant barrier declined from 21.0% in 2017 to 3.3% in 2021 (Fisher’s exact *p* = .016).

**Fig. 4:**
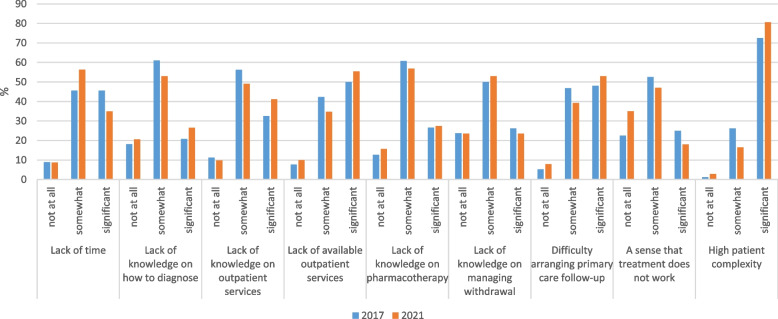
Barriers to caring for SUD in inpatients, change over time among nurses (*n* = 184)^a^. ^a^Fisher’s exact tests were used to examine differences by year; all ns (*p* > .05)

Nurses likewise identified high patient complexity as the top barrier to caring for SUD in inpatients, with 72.5 and 80.6% reporting it to be a significant barrier in 2017 and 2021, respectively (Fig. 4). Sizable proportions reported a lack of available outpatient services (50.0% in 2017 and 55.4% in 2021) and difficulty arranging primary care follow-up (48.1% in 2017 and 52.9% in 2021). There was no significant change in nurses’ reports of barriers over time.

**Fig. 5:**
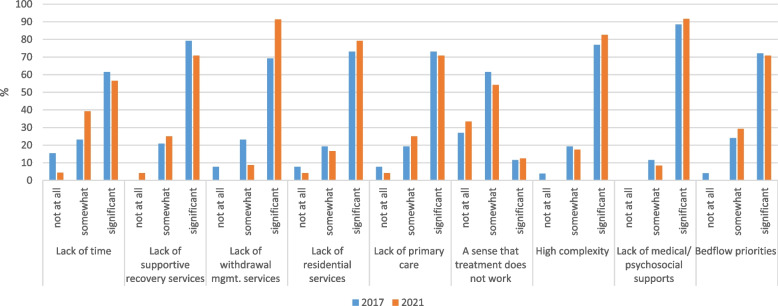
Barriers to caring for SUD in inpatients, change over time among social workers (*n* = 50)^a^. ^a^Fisher’s exact tests were used to examine differences by year; all ns (*p* > .05)

Relative to nurses and physicians, social workers endorsed a greater number of barriers to caring for SUD in inpatients (Fig. 5). Across both time points, the following were reported as barriers by over half of respondents: lack of time (61.5% in 2017, 56.5% in 2021), supportive recovery services (79.2, 70.8%), withdrawal management services (69.2, 91.3%), residential care (73.1, 79.2%), primary care (73.1, 70.8%), medical/psychosocial supports (88.5, 91.7%), high patient complexity (76.9, 82.6%) and bed flow priorities (72.0, 70.8%). There were no significant changes in social workers’ reports of barriers over time.

Respondents had the opportunity to describe additional barriers encountered in providing quality care to people with SUD. Many elaborated on challenges related to the barriers summarized above (e.g., high patient complexity commonly involving co-occurring mental disorders, lack of community supports including outpatient treatment and housing). Additional themes emerged related to substance-related stigma among health care providers (e.g., “*the belief among many health care workers that all users can simply choose to stop using, ‘if they cared enough’ to improve their health, is a significant barrier to accessing appropriate and respectful care for inpatients with substance use disorders.” SW-2017)*; challenges related to managing behaviours and engaging people in care while they are in a hospital setting (e.g., unpredictable behaviours, non-compliance, violence); and barriers related to existing care processes, including cumbersome referral processes and heavy patient volume coupled with the time required to engage people with SUD (e.g., “*On surgical units its such a rush to discharge people as there are always need for beds for post ops coming in. Often times I find when patients have underlying substance issues they often have felt pushed out of hospital and like a burden for wanting to stay longer for treatment.” N-2021).* Also relevant to these process-related barriers, a number of nurses mentioned challenges connecting with physicians (e.g., *“ability to communicate with MRP [most responsible physician] providers regularly, increased time and effort when needing to page or try and get ahold of MRP can lead to issues with patient care and patients wanting to leave AMA [against medical advice]” N-2021*).

## Discussion

In this study, we sought to evaluate provider awareness of the service and changes over time in their perceptions of care for inpatients with SUD. Four years in, participants were aware and reasonably familiar with the service, and had positive perceptions of its impact. Providers reported that they frequently encountered SUD and related conditions in their practice, but encountered significant barriers in meeting the needs of these patients. Findings point to an improvement in perceived quality of inpatient care over time, with room left for further enhancement of the AMCS and the broader regional system of care.

In particular, we found room for improvement in supporting nurses to care for inpatients with SUD. Relative to physicians and social workers, nurses were less likely to be familiar with the AMCS, know how to access it, or see it as beneficial. This discrepancy may reflect that nurses are not directly involved in requesting AMCS consults and do not have a direct line of communication with the AMCS. At present, the AMCS team in Victoria does not include a nurse liaison. Elsewhere, AMCS teams have integrated nurses to support patient care and to liaise with fellow nursing staff [13, 17]. Outreach, education, and other efforts are warranted to ensure that nurses feel supported and confident caring for this patient population. Prior research has shown that beyond educational efforts (e.g., grand rounds, symposia, and nurse educator positions), dedicated nursing role support (e.g., access to nursing expertise for advice and assistance) is critical to increasing knowledge, reducing stigma, and ensuring consistent quality care [20, 24].

Despite perceived increases in care quality (most notably among physicians), there was little change in levels of comfort in addressing SUD. Elsewhere, AMCS teams have participated in educational and inter-professional initiatives to support system-based dissemination of best practices in hospital-based SUD care and increasing overall provider comfort with managing SUD [13, 25]. By implementing practices to decentralize medical knowledge, specialists may provide expertise and support their colleagues in delivering high-quality specialized care to patients with SUD within their own scope of practice.

Providers also reported persistent barriers to caring for inpatients with SUD related to patient complexity and connecting people to community services of all kinds. This was particularly true for social workers, who facilitate connections to community services pre- and post-discharge. Prior research highlights the importance of bridging the gap between hospital and outpatient care, with patients describing frustration with long wait times for treatment after discharge from hospital [12]. Strong community partnerships, active in-hospital referrals, and rapid-access pathways are critical to optimizing the effectiveness of AMCS teams [12, 18]. This is feasible only to the extent that there is a strong community sector, including coordinated harm reduction services, withdrawal management, counselling-based interventions, pharmacology, primary care, and supports for housing [26]. Interventions to link inpatients with community supports require a system with capacity to meet the needs of the population. In under-resourced settings (e.g., hospitals and communities without addiction specialists or access to dedicated facilities), optimization prior to discharge is key; with tools available for non-specialist providers such as clinical checklists outlining treatment options for infection prevention, screening, and harm reduction [27].

This study took place during dual declared public health emergencies in British Columbia, related to COVID-19 and illicit drug overdoses. The pandemic has resulted in changing patterns of substance use in the population, as well as disruptions to income and housing supports, community and hospital based care for SUD and other health conditions [28–30]. Many AMCS teams have adapted with changes in mode of service delivery (e.g., telehealth), prescribing practices (e.g., length of bridge prescriptions at discharge), dissemination of COVID-19 specific harm reduction guidelines, and increased peer support and social work outreach following discharge [31]. This context is likely to have affected providers’ perceptions of care and barriers to community services. It remains to be seen if efforts to enhance the regional system of care for people with SUD, including but not limited to development of the AMCS, will result in longer-term improvements in hospital and community based services, their coordination, and subsequent health outcomes.

In this study, we were successful in capturing a large sample of inpatient care providers. Further research using semi-structured interviews or focus groups would likely yield valuable insights on the complexity of care delivery for this population, to complement findings from this survey. Such work would help clarify any potential changes in perceived quality of care over time, given the potential for over-sensitivity of the 10-point measurement scale used in the present study [32]. Future work is also needed to capture patient perspectives on their care, their experiences of the transition from hospital to community, and health outcomes. Limitations of the current study include the non-random (convenience) sample. Given the passive recruitment strategy, we are unable to comment on the number of potential respondents who opted not to participate. As such, findings may not generalize to all providers within this or similar settings. Given the context of the dual public health emergencies and the uncontrolled study design, we cannot attribute changes in provider perceptions of SUD care quality to the AMCS specifically. Nonetheless, findings offer a valuable look into AMCS implementation and provide insights into potential improvements for the future.

## Conclusions

This study provides valuable insights into the process of establishing an inpatient AMCS in a small urban setting. Findings indicate that the AMCS is filling a gap that existed previously in the local system of care; however, numerous opportunities exist to further strengthen the system beyond the hospital setting to promote longer-term health among people who use substances. When establishing a new AMCS in a general hospital setting, efforts should be explored to ensure that providers are adequately supported to care for people with SUD (such as through outreach, education, and dedicated liaison positions). Accompanying enhancement of community systems may be particularly needed outside of large urban settings, where services and supports may be less available. In order to maximize the effectiveness of an inpatient AMCS, seamless transition to community services and supports is critical.

## Data Availability

The datasets used and analysed during the current study are available from the corresponding author on reasonable request.

## Abbreviations

AMCS: Addiction medicine consult service
IV: Intravenous
SUD: Substance use disorder
